# Regulation of Endothelial Cell Adhesion Molecule Expression by Mast Cells, Macrophages, and Neutrophils

**DOI:** 10.1371/journal.pone.0014525

**Published:** 2011-01-14

**Authors:** Jie Zhang, Pilar Alcaide, Li Liu, Jiusong Sun, Aina He, Francis W. Luscinskas, Guo-Ping Shi

**Affiliations:** 1 Department of Medicine, Harvard Medical School, Brigham and Women's Hospital, Boston, Massachusetts, United States of America; 2 Department of Pathology, Harvard Medical School, Center for Excellence in Vascular Biology, Brigham and Women's Hospital, Boston, Massachusetts, United States of America; 3 School of Life Sciences, Huzhou Teachers College, Huzhou, China; Universität Würzburg, Germany

## Abstract

**Background:**

Leukocyte adhesion to the vascular endothelium and subsequent transendothelial migration play essential roles in the pathogenesis of cardiovascular diseases such as atherosclerosis. The leukocyte adhesion is mediated by localized activation of the endothelium through the action of inflammatory cytokines. The exact proinflammatory factors, however, that activate the endothelium and their cellular sources remain incompletely defined.

**Methods and Results:**

Using bone marrow-derived mast cells from wild-type, *Tnf^−/−^*, *Ifng^−/−^*, *Il6^−/−^* mice, we demonstrated that all three of these pro-inflammatory cytokines from mast cells induced the expression of vascular cell adhesion molecule-1 (VCAM-1), intercellular adhesion molecule-1 (ICAM-1), P-selectin, and E-selectin in murine heart endothelial cells (MHEC) at both mRNA and protein levels. Compared with TNF-α and IL6, IFN-γ appeared weaker in the induction of the mRNA levels, but at protein levels, both IL6 and IFN-γ were weaker inducers than TNF-α. Under physiological shear flow conditions, mast cell-derived TNF-α and IL6 were more potent than IFN-γ in activating MHEC and in promoting neutrophil adhesion. Similar observations were made when neutrophils or macrophages were used. Neutrophils and macrophages produced the same sets of pro-inflammatory cytokines as did mast cells to induce MHEC adhesion molecule expression, with the exception that macrophage-derived IFN-γ showed negligible effect in inducing VCAM-1 expression in MHEC.

**Conclusion:**

Mast cells, neutrophils, and macrophages release pro-inflammatory cytokines such as TNF-α, IFN-γ, and IL6 that induce expression of adhesion molecules in endothelium and recruit of leukocytes, which is essential to the pathogenesis of vascular inflammatory diseases.

## Introduction

Leukocyte adhesion and transendothelial migration are important components of atherogenesis and other inflammatory vascular diseases. Leukocyte recruitment is a multi-step process that typically consists of: leukocyte initial tethering and rolling on the surface of activated endothelial cells (EC), leukocyte arrest and firm adhesion, and subsequent transmigration to the neointima or site of inflammatory tissues. The initial rolling process of leukocytes is mediated mainly by the selectins expressed by activated endothelium [1] and selectin ligand PSGL-1 (P-selectin glycoprotein ligand-1) on the leukocyte surface [2], whereas leukocyte arresting and firm adhesion is mediated primarily by vascular cell adhesion molecule-1 (VCAM-1) and intercellular adhesion molecule-1 (ICAM-1) on the endothelium that bind β1 and β2 integrins expressed on leukocytes [1]–[4]. Endothelium overlaying human atherosclerotic lesions and fatty streak, or from patients with unstable angina, express high levels of E- and P-selectins [5], [6], ICAM-1 and VCAM-1 [7], [8]. PSGL-1 monoclonal antibodies abolish most leukocyte rolling [9], and selectin deficiency reduces leukocyte recruitment and atherosclerosis [10], [11]. VCAM-1 is one of the earliest markers of atherosclerotic lesions in animal models and thus a key adhesion molecule mediating leukocyte recruitment to early lesions [12], [13]. EC transfected with VCAM-1 support monocyte rolling and firm adhesion [14]. Antibodies blocking either VCAM-1 or the β1 or β2 integrins greatly reduce monocyte adhesion [15], and genetic mutation of ICAM-1 or VCAM-1 reduces atherosclerosis in mice [3], [13], [16]. Therefore, expression of these endothelial adhesion molecules strongly influences the progression of atherosclerosis.

In addition to using PSGL-1 and integrins for rolling and adhesion, leukocytes are also rich sources of pro-inflammatory cytokines. TNF-α, IFN-γ, IL1, and IL4 are well-characterized stimuli that induce endothelial cell adhesion molecule expression [12], [17]–[19] and promote leukocyte adhesion and recruitment [20]. However, these in vitro experiments were performed using purified cytokines, and each of these leukocyte subsets may produce an overlapping sets of cytokines. Therefore, it remains unknown which types of inflammatory cells are more important in cytokine production and induction of EC adhesion molecule expression and leukocyte recruitment. It is possible that all inflammatory cells — including monocytes, neutrophils, lymphocytes, and mast cells — contribute to the pool of cytokines, and thus are equal in enhancing adhesion molecule expression and leukocyte infiltration. In addition to cytokines, leukocytes release other molecules that affect endothelial adhesion molecule expression through different mechanisms. For example, mast cell-derived histamine, neutrophil elastase, tryptase, substance P, and angiotensin-II increase endothelial adhesion molecule expression and leukocyte rolling, adhesion, and emigration. Pre-treatment with function blocking monoclonal antibodies to adhesion molecules [21] or cytokines [22] reduces these leukocyte responses.

We recently demonstrated that mast cells are essential cellular components in atherosclerosis. Mast cell–deficient (*Kit^W-sh/W-sh^*) mice were protected from diet-induced atherosclerosis [23]. Atherosclerotic lesions in these mice contained fewer macrophages and T-cells, suggesting reduced leukocyte recruitment occurred in the absence of mast cells [22]. In a separate study, we used the same mice and tested the role of mast cells in white adipose tissue growth. Deficiency or inactivation of mast cells reduced body weight gain and adipose tissue macrophage infiltration, suggesting that mast cells play a role in obesity and in recruiting macrophages, although the exact molecules responsible for macrophage infiltration were not examined [24]. In this study, we tested the effects of mast cells, neutrophils, and macrophages from TNF-α-, IFN-γ- and IL-6-deficient mice on mouse heart EC (MHEC) adhesion molecule expression and leukocyte adhesion. Our results demonstrated that all tested inflammatory cells use TNF-α for MHEC adhesion molecule expression, although IL6 and IFN-γ had different levels of contributions to MHEC activation.

## Materials and Methods

### Ethics statement

The Harvard Medical School Institutional Animal Care and Use Committee approved this animal protocol (Protocol number 03579).

### Mice and reagents

Male wild-type mice (C57BL/6 background), *Tnf^−/−^* (backcrossed two generations [N2] into C57BL/6 background), *Ifng^−/−^* (C57BL/6, N10), and *Il6^−/−^* (C57BL/6, N11) mice were purchased from the Jackson Laboratory (Bar Harbor, ME). *Tnf^−/−^* mice were further backcrossed into congenic C57BL/6 background (N10). Anti-PECAM-1 antibody and anit-ICAM-2 antibody were purchased from Pharmingen (San Diego, CA). Percoll, calcium ionophore A23187, and Sybr Green were purchased from Sigma (St. Louis, MO). Mouse VCAM-1, ICAM-1, P-selectin, and E-selectin ELISA kits were purchased from R&D Systems (Minneapolis, MN).

### Mouse endothelial cell isolation

Mouse heart endothelial cells (MHEC) were prepared from collagenase treated myocardial tissue, using a two step positive selection protocol with anti-CD31 and anti-ICAM-2 coated magnetic beads, as described [25]. A slight modification of the published protocol was the use of hearts from newborn animals (7–9 days old), which yielded cells that more consistently formed uniform monolayers amenable to analysis of events by DIC microscopy [25]. MHEC were cultured in high glucose-DMEM medium with 20% heat-inactivated fetal calf serum (FCS), 100 U/mL penicillin, 100 ug/mL streptomycin, 250 ng/mL fungizone and 10 ng/mL endothelial cell growth factor. All MHEC were used in these experiments were used at subculture two.

### Mast cell isolation and degranulated mast cell supernatant preparation

Bone marrow-derived mast cells (BMMC) were differentiated from bone marrow cell preparation in the presence of IL3 and stem cell factor (SCF), as described previously [23], [24]. In brief, bone marrow cells from mouse femurs and tibias were flushed with RPMI 1640 and cultured in RPMI 1640 with 10% FCS, 50 µM of 2-mercaptoethanol and 20 ng/mL of IL3 (PeproTech, Rocky Hill, NJ). After two weeks, 20 ng/mL of SCF (PeproTech, Rocky Hill, NJ) was added to the BMMC culture medium. Cells were cultured for additional four weeks in IL3 and SCF.

To degranulate mast cells, we cultured BMMC (3×10^6^/mL) in RPMI 1640 with a mast cell secretagogue, calcium ionophore A23187 (500 ng/mL, Sigma) for six hours at 37°C, as previously described [26]. Degranulated mast cell supernatants were aliquoted and stored at −80°C and used to activate MHEC.

### Neutrophil and macrophage isolation

Mouse neutrophils were prepared from bone marrow using Percoll gradient (62.5%, Sigma) centrifugation, according to a previously reported method [27]. Peritoneal macrophages were prepared by intraperitoneal elicitation of mice with 1 mL of a 3% thioglycollate media (Sigma). Two days later, mouse peritoneal cavity fluids were flushed with 1xPBS containing 1% FCS and 10 mM EDTA, centrifuged, and macrophages resuspended in cold DMEM. Neutrophil and macrophage cell lysates were prepared by sonicating cells (3×10^6^/mL media) in DMEM containing 10% FCS. Macrophage conditioned media were also prepared by incubating 3×10^6^/mL cells in DMEM with 500 ng/mL A23187, as we did for mast cells. Cell lysates and conditioned media were collected and stored at −80°C for consequent MHEC culture.

### Enzyme-linked immnosorbent assay (ELISA)

Confluent mouse MHEC monolayers in a six-well plate were incubated at 37°C in DMEM complete medium for 24 hours with degranulated BMMC supernatants from different gene knockout mice (1∶10 dilution in DMEM). Treatment with DMEM alone with same amount of A23187 (50 ng/mL) was used as experimental control. The MHEC monolayers were washed with ice-cold 1xPBS briefly and lysed in lysis buffer containing 25 mM Tris-HCl pH 7.6, 150 mM NaCl, and 1% Triton X-100. Protein concentration was determined using Bio-Rad protein assay. One microgram of total protein from each well was used for ELISA to determine EC adhesion molecule protein levels, according to the manufacturer's instructions (DuoSet ELISA Development System, R&D Systems).

IL6, IFN-γ, and TNF-α levels in mast cell conditioned media and in macrophage and neutrophil lysates at 3×10^6^ cells per mL media prepared from WT and different cytokine-deficient mice were determined with ELISA (Aushon BioSystems, Inc., Billerica, MA).

### Real time-polymerase chain reaction (RT-PCR)

Mouse MHEC monolayers were cultured on a gelatin-coated six-well plate in 2 mL of DMEM complete medium at 37°C. At 100% confluence, culture medium was replaced by 1.8 mL of fresh MHEC culture medium containing 0.2 mL of degranulated supernatant from mast cells, neutrophils or macrophages, giving cellular proteins from the same numbers cells (3×10^5^ cells/mL of supernatant). MHEC were harvested 4 hrs later.

Total cellular RNA of MHEC was extracted using TRIzol reagent (GIBCO-BRL) and treated with RNase-free DNase (DNase I, Ambion, Inc., Austin, TX) to remove potential genomic DNA contaminants. One microgram total RNA was reverse-transcribed with RETROscript™ two-step RT-PCR system (Ambion, Inc.). Quantitative PCR was performed in a single-color RT-PCR detection system (Stratagene, Santa Clara, CA). Gene expression level (mRNA) of each adhesion molecule was normalized to that of β-actin transcript. The primer sequences of VCAM-1, ICAM-1, P-selectin, and E-selectin were as follows: VCAM-1 (f) 5′-AGTTGGGGATTCGGTTGTTCT-3′; VCAM-1 (r) 5′-CCCCTCATTCCTTACCACCC-3′; ICAM-1 (f) 5′-GTGATGCTCAGGTATCC ATCCA-3′; ICAM-1 (r) 5′-CACAGTTCTCAAAGCACAGCG-3′; P-selectin (f) 5′-CATCTGGTTCAGTGCTTTGATCT-3′; P-selectin (r) 5′-ACCCGTGAGTTATTC CATGAGT-3′; E-selectin (f) 5′-ATGCCTCGCGCTTTCTCTC-3′; E-selectin (r) 5′-GTAGTCCCGCTGACAGTATGC-3′.

### Measurements of neutrophil adhesion to activated endothelium under physiological shear flow

MHEC were grown to confluence on 25-mm^2^ glass coverslips (Carolina Biological Supply, Burlington, NC) pre-coated with 5 µg/mL of fibronectin (Sigma). MHEC monolayers were stimulated with degranulated mast cell supernatants prepared from BMMC from WT, *Tnf* ^−/−^, *Ifng* ^−/−^, and *Il6* ^−/−^ mice for 16 hours. Culture medium alone was used as a negative control. TNF-α (25 ng/mL; 16 hr) in culture medium was used as a positive control. Coverslips were inserted into the flow chamber and freshly isolated neutrophils (5×10^5^/mL) suspended in a flow buffer (1xPBS with 0.1% human serum albumin, Baxter Healthcare, Glendale, CA) were drawn across the endothelial surface at a low shear stress (0.5 dyne/cm^2^). Cell adhesion was determined after the initial minute of each flow rate by counting the number of adherent cells in eight different fields, recorded using a video microscopy system [28].

### Statistical analysis

We used Student's *t* test to examine the differences between the groups. *P*<0.05 was considered statistically significant.

## Results

### MHEC adhesion molecule expression regulation by mast cells

**Figure 1:**
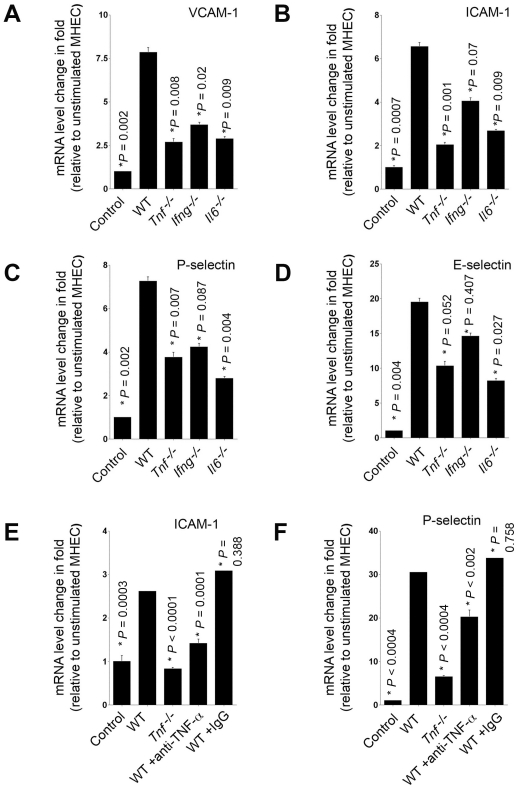
Degranulated mast cells induced MHEC adhesion molecule expression. RT-PCR determined VCAM-1 (A), ICAM-1 (B), P-selectin (C), E-selectin (D), ICAM-1 (E), and P-selectin (**F**) mRNA levels relative to β-actin in MHEC after stimulation with conditioned media of mast cells from WT and different cytokine-deficient mice, or WT mast cell conditioned media pre-treated with anti-mouse TNF-α antibody or isotype control IgG (R&D Systems). Fold change was calculated relative to unstimulated MHEC. Data were presented as mean ± SE of three independent experiments. *P*<0.05 was considered statistically significant, Student's *t* test. *Compared with WT BMMC-treated MHEC.

Cytokines in recombinant or purified forms are potent inducers of EC adhesion molecule expression in vitro. To examine the role of specific mast cell–derived cytokines in EC adhesion molecule expression, we prepared degranulated mast cell supernatants from knockout mice that were genetically deficient in the three main mast cell cytokines — IL-6, TNF-α, and IFN-γ — and cocultured these degranulated supernatant preparations to stimulate freshly prepared MHEC monolayers. Cytokine ELISA demonstrated that deficiency of one cytokine showed compensatory increase of other cytokines at different levels in mast cell conditioned media, in which IL6 had the highest levels followed by TNF-α and IFN-γ (Table 1). After four hours of incubation, WT MHEC stimulated with degranulated mast cell preparation from WT mice expressed significantly higher mRNA levels of VCAM-1, ICAM-1, P-selectin, and E-selectin than those treated with culture media alone, as determined by RT-PCR (Figure 1A–1D). This finding is consistent with earlier studies [26]. However, when degranulated mast cell supernatants from *Il6^−/−^* or *Tnf^−/−^* mice were tested, MHEC expressed significantly lower levels of VCAM-1, ICAM-1, P-selectin, and E-selectin than those treated with WT mast cells (Figure 1A-1D). In contrast, supernatants of mast cells from *Ifng^−/−^* mice showed lower induction of MHEC adhesion molecule expression as compared to WT supernatants; but only VCAM-1 expression reached statistical significance (Figure 1A). These observations suggest that mast cell–derived TNF-α, IL6, and IFN-γ are potent stimuli that induce EC VCAM-1, ICAM-1, P-selectin, and E-selectin expression, although IFN-γ is the weakest of the three.

**Table 1:** Cytokine levels in mast cell conditioned media and macrophage and neutrophil lysates used for MHEC stimulation.

Cell type	IL6 (pg/mL)	IFN-γ (pg/mL)	TNF-α (pg/mL)
Mast cell			
WT	221,685.9	11.2	1813.3
*Il6^−/−^*	–	360.9	3136.3
*Ifng^−/−^*	340,844.2	–	2109.7
*Tnf^−/−^*	381,912.1	35.3	–
Macrophage			
WT	9.4	2.1	2.6
*Il6^−/−^*	–	2.2	3.7
*Ifng^−/−^*	7.0	–	1.7
*Tnf^−/−^*	5.2	2.1	–
Neutrophil			
WT	5.1	7.8	6.1
*Il6^−/−^*	–	7.8	4.1
*Ifng^−/−^*	6.2	–	5.2
*Tnf^−/−^*	2.6	7.8	–

To affirm a role of mast cell-derived cytokines in MHEC adhesion molecule expression, we pre-incubated mast cell conditioned media from WT mice with an anti-mouse TNF-α antibody (25 µg/mL, R&D Systems) for 2 hours at 37°C. Similar to the conditioned media from *Tnf^−/−^* mast cells, anti-TNF-α antibody-treated, but not isotype control IgG-treated WT mast cell conditioned media also showed significant reductions of ICAM-1 and P-selectin mRNA levels in MHEC (Figure 1E/1F).

**Figure 2 pone-0014525-g002:**
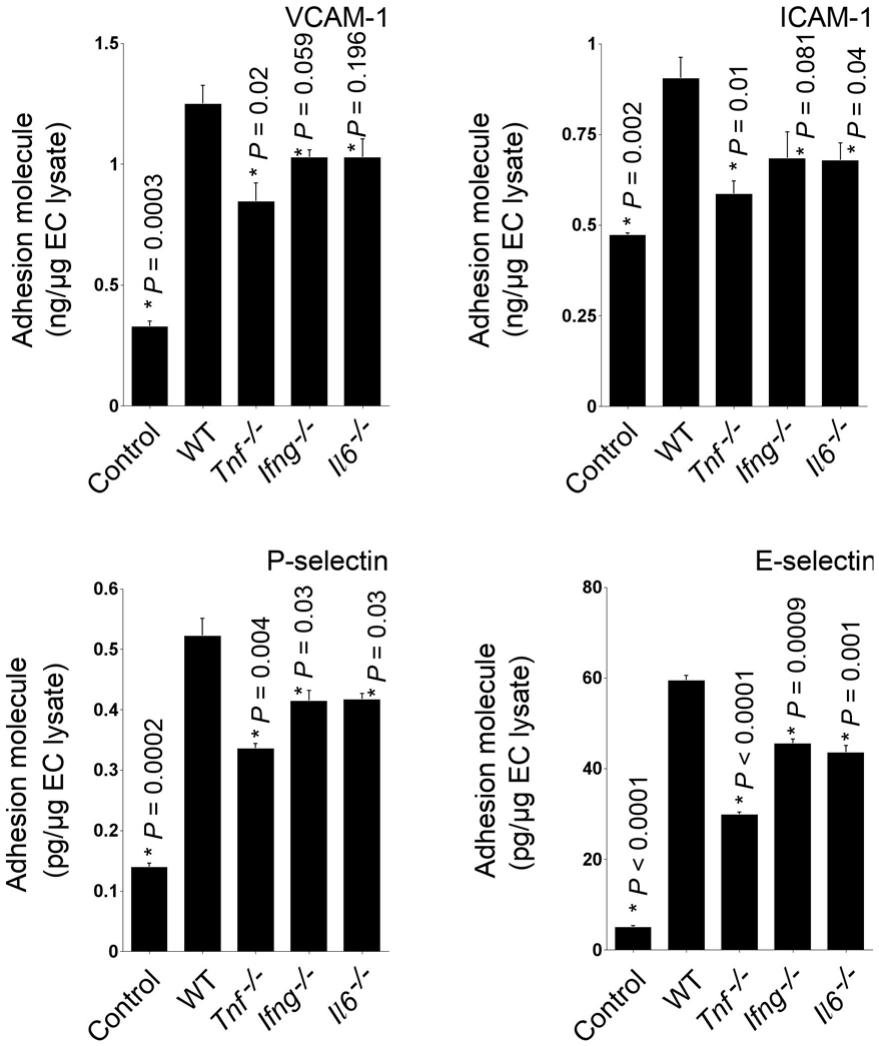
Adhesion molecule protein levels from MHEC treated with different degranulated mast cell supernatants. Commercial ELISA kits were used to determine VCAM-1, ICAM-1, P-selectin, and E-selectin in MHEC lysate. Degranulated mast cell supernatants from different mice, as indicated, were used to treat mouse MHEC for 24 hours. Data are presented as mean ± SE of three independent experiments. *P*<0.05 was considered statistically significant, Student's *t* test. *Compared with WT BMMC-treated MHEC.

Similar results were found when MHEC lysates were assessed after 24 hours of incubation by ELISA. Degranulated WT mast cell supernatants stimulated the protein levels of all four tested adhesion molecules in MHEC. In contrast, supernatants from individual *Tnf^−/−^*, *IL6^−/−^*, or *Ifng^−/−^* mice showed significantly reduced induction of P-selectin and E-selectin protein production (Figure 2). MHEC protein levels of VCAM-1 and ICAM-1, however, were slightly different from the effects on selectin expression. Mast cell supernatants from *Tnf^−/−^* showed impaired induction of both VCAM-1 and ICAM-1; mast cells from *Ifng^−/−^* mice were not significantly different from WT mast cells in VCAM-1 and ICAM-1 expression, and mast cells from *Il6^−/−^* mice showed significant reduction in ICAM-1 protein levels but not in VCAM-1 (Figure 2). Therefore, TNF-α is an important cytokine produced by mast cells in the induction of MHEC adhesion molecule expression. IL6 and IFN-γ from mast cells are essential in MHEC selectin expression, but are less potent in VCAM-1 and ICAM-1 protein expression.

### Mast cell-derived cytokines in neutrophil adhesion

**Figure 3:**
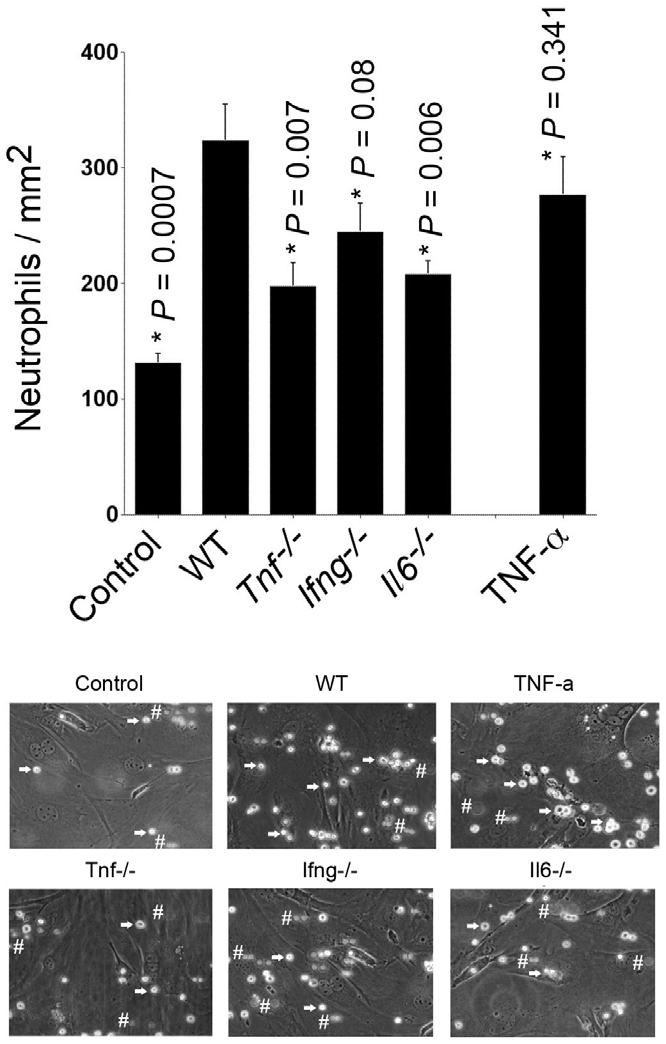
Neutrophil adhesion assay on activated MHEC monolayers under physiological shear stress. MHEC monolayer was activated with different degranulated mast cell supernatants for 16 hours and then examined for adhesion of bone marrow neutrophils from WT mice under shear flow conditions of 0.5 dynes/cm^2^. Neutrophil adhesion on MHEC monolayers was quantified as reported previously. Culture media alone was used as a negative control and recombinant TNF-α (25 ng/mL) was used as a positive control. Data were the mean ± SE from eight different fields. *P*<0.05 was considered statistically significant, Student's *t* test. *Compared with WT BMMC-treated MHEC. Representative photographs captured from videos are shown in the bottom panels. Arrows indicate examples of adhered cells bound to MHEC. Cells that are not adhered to MHEC are flowing by, therefore they are out of focus and not as bright as adhered cells: Examples are indicated with #.

Increased adhesion molecules in MHEC after coincubation with supernatants from degranulated mast cells and reduced induction of adhesion molecule expression in MHEC when cytokine-deficient mast cells were used suggest that mast cells are an important source of inflammatory products that enhance leukocyte adhesion, and this mast cell function is associated, in part, with mast cell cytokines. To examine this hypothesis further, we assessed neutrophil adhesion to co-cultured MHEC monolayers under conditions of low shear stress (0.5 dynes/mm^2^) [28]. Consistent with the MHEC adhesion molecule expression patterns, degranulation supernatants from WT mast cells increased neutrophil adhesion to WT MHEC monolayer more than twofold over those treated with medium alone, which was as potent as the recombinant TNF-α alone positive control (Figure 3). In contrast, mast cells from *Tnf^−/−^* and *Il6^−/−^* mice demonstrated significant reduction in the ability to enhance neutrophil adhesion. Compared to MHEC treated with WT mast cell supernatants, neutrophil adhesion to *Ifng^−/−^* mast cell–activated MHEC monolayer was also reduced, but did not reach statistical significance (Figure 3). Our data from this shear stress flow model suggest that mast cell–derived TNF-α and IL6 contribute to leukocyte adhesion during the pathogenesis of atherosclerosis or other vascular diseases.

### Macrophage-derived cytokines in MHEC adhesion molecule expression

**Figure 4:**
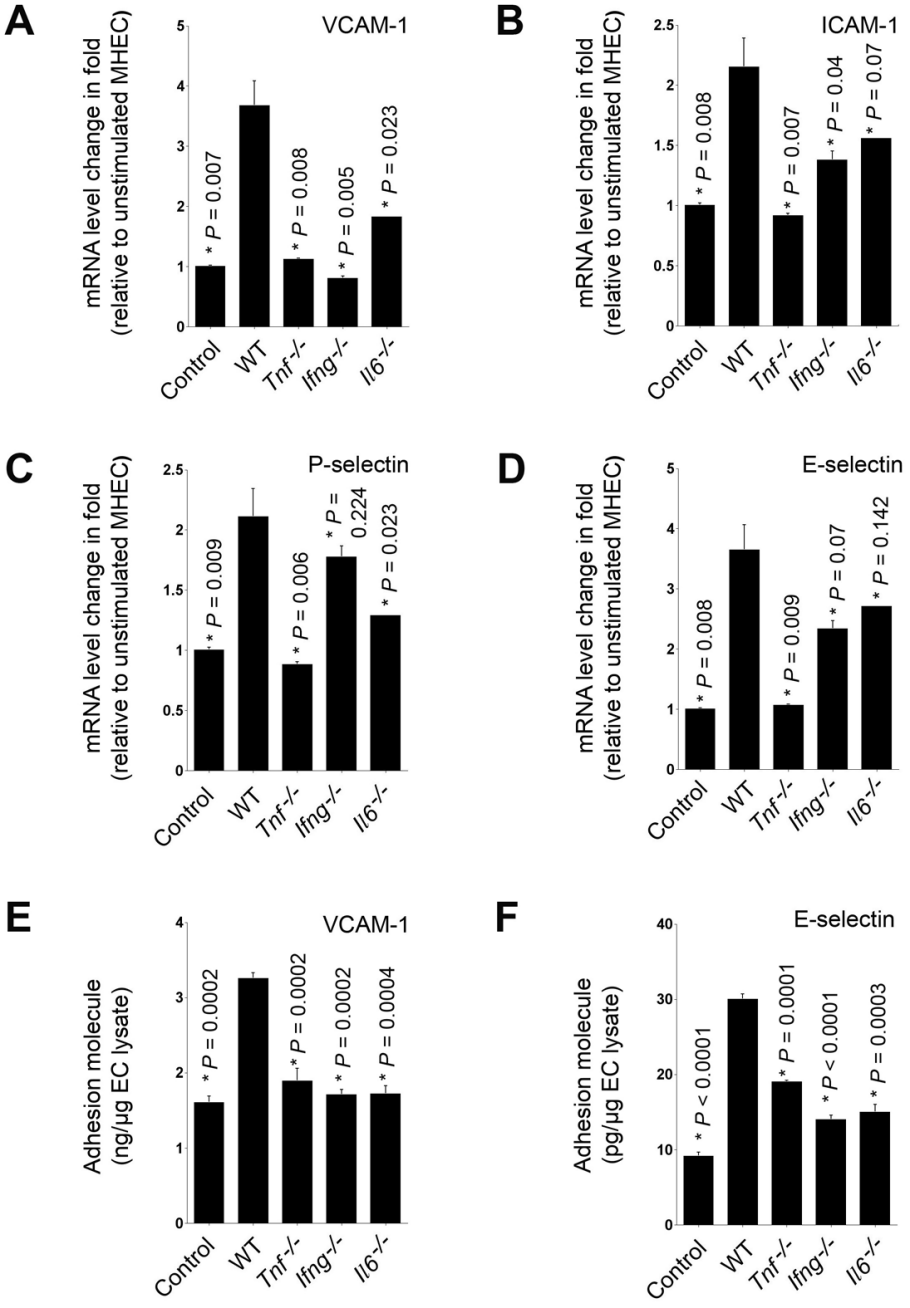
Adhesion molecule expression in MHEC after macrophage stimulation. MHEC were treated with macrophage cell lysates from different mice, as indicated, for four hours. VCAM-1 (**A**), ICAM-1 (**B**), P-selectin (**C**), and E-selectin (**D**) mRNA levels were quantified by RT-PCR and normalized to β-actin. Fold changes were calculated relative to unstimulated EC. ELISA determined VCAM-1 (**E**) and E-selectin (**F**) protein levels in lysates of MHEC that were stimulated with macrophage lysates from different mice for 24 hours. Data were presented as mean ± SE of three to six independent experiments. *P*<0.05 was considered statistically significant, Student's *t* test. *Compared with MHEC treated with WT macrophage lysate.

Before the initiation of leukocyte rolling and adhesion, all leukocytes have an equal opportunity to affect the endothelium in the venular wall. As one of the most abundant tissue inflammatory cells, macrophage may also produce cytokines to stimulate MHEC adhesion molecule expression and enhance their own adhesion as well as adhesion of other leukocyte types. To test this hypothesis, we prepared macrophage conditioned media by incubating macrophages with calcium ionophore A23187 as we did for mast cells. MHEC stimulated with macrophage conditioned media from WT, *Tnf^−/−^*, *Ifng^−/−^*, and *Il6^−/−^* mice showed no significant differences in mRNA levels of VCAM-1, ICAM-1, P-selectin, and E-selectin from unstimulated MHEC (data not shown). Thus, we treated mouse MHEC monolayers with macrophage lysates prepared from different cytokine-deficient mice. Similar to our results with mast cells, lysates from WT macrophages increased the mRNA levels of all four adhesion molecules in MHEC, although macrophage lysates contained lower levels of IL6, TNF-α, and IFN-γ (Table 1) and induced lower levels of VCAM-1, ICAM-1, P-selectin, and E-selectin mRNA (Figure 4A–4D) than did mast cell conditioned media (Figure 1A–1D). In contrast, macrophage lysates from *Tnf^−/−^* mice did not exhibit this effect (Figure 4A–4D), demonstrating an important role of macrophage-derived TNF-α in EC adhesion molecule expression. Absence of IFN-γ macrophages completely blocked their ability to induce VCAM-1 expression in MHEC, and significantly impaired ICAM-1 expression as compared to WT macrophages (Figure 4A/4B). The same macrophage lysates showed much weaker activities in inducing MHEC selectin expression. Unlike in mast cells, macrophage lysates from IL6-deficient mice were a weak inducer of all four adhesion molecules. The absence of IL6 in macrophages reduced significantly the induction of VCAM-1 and P-selectin expression as compared with WT macrophages, but did not have a significant effect on expression of ICAM-1 or E-selectin (Figure 4A–4D). Different from the mRNA levels, protein levels of adhesion molecules, such as VCAM-1 and E-selectin, were all significantly lower in MHEC treated with macrophage lysates from *Tnf^−/−^*, *Ifng^−/−^*, and *Il6^−/−^* mice than in WT macrophage lysate-treated MHEC (Figure 4E/4F). These data strongly demonstrate that macrophages are an important source of inflammatory cytokines that induce expression of endothelial selectins and VCAM-1 and ICAM-1.

### Neutrophil effect in MHEC adhesion molecule expression

**Figure 5:**
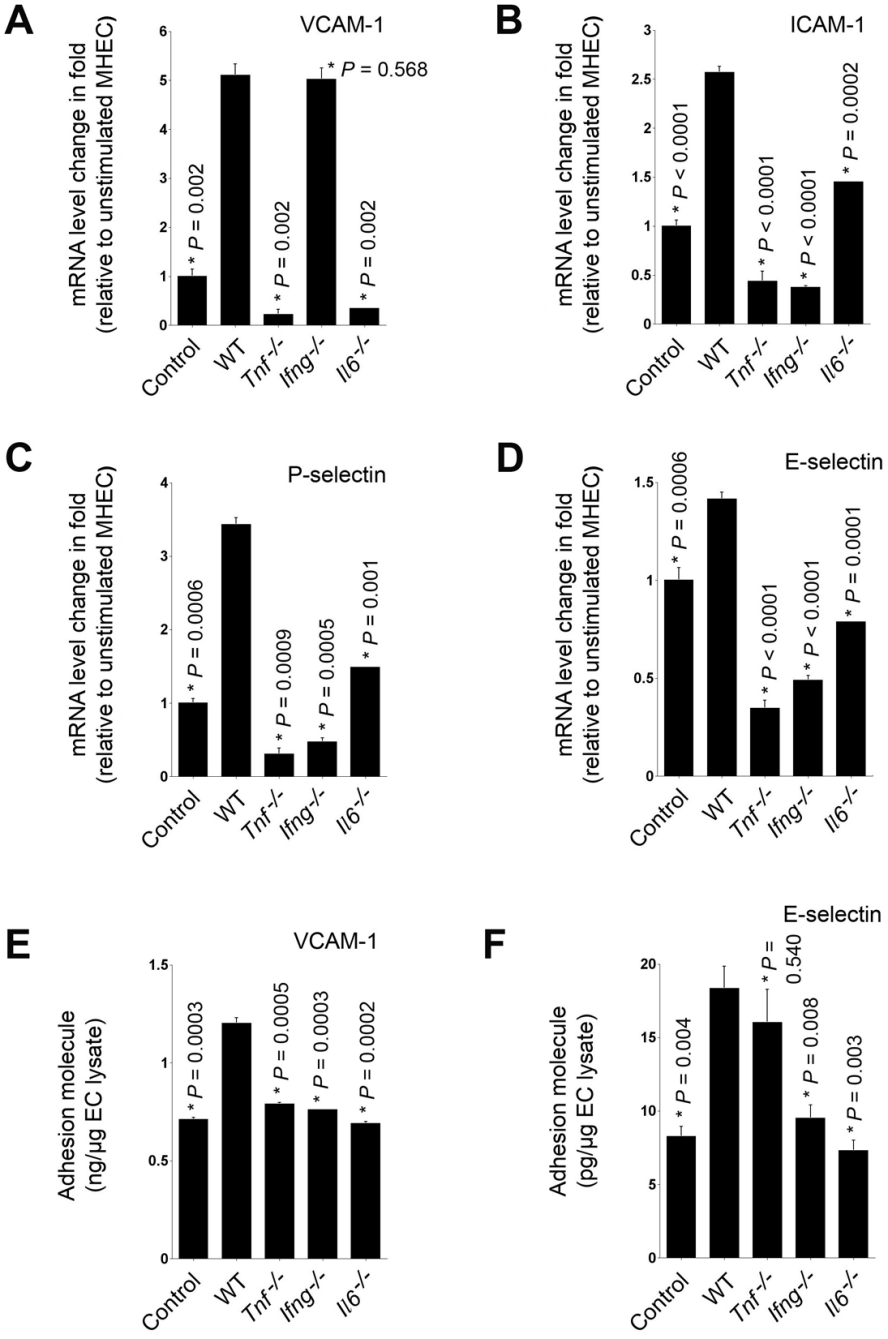
Adhesion molecule expression in MHEC after neutrophil stimulation. Neutrophil lysates were prepared from bone marrow from different mice, as indicated, and used to induce MHEC for four hours. VCAM-1 (**A**), ICAM-1 (**B**), P-selectin (**C**), and E-selectin (**D**) mRNA levels were quantified by RT-PCR and normalized to β-actin. Fold changes were determined relative to unstimulated MHEC. ELISA determined VCAM-1 (**E**) and E-selectin (**F**) protein levels in lysates of MHEC that were stimulated with neutrophil lysates from different mice, as indicated, for 24 hours. Data were presented as mean ± SE of three to six independent experiments. *P*<0.05 was considered statistically significant, Student's *t* test. *Compared with MHEC treated with WT neutrophil lysate.

Neutrophils are the most abundant leukocytes in the peripheral blood. Human atherosclerotic lesions — especially ruptured lesions or lesions from patients with unstable angina pectoris — contain high numbers of neutrophils [29], [30]. In murine atherosclerotic lesions, neutrophils appear in the adventitia, fibrous cap, and shoulder, and at some sites, they colocalize with P-selectin–positive endothelium [31]–[33]. In some cases, neutrophils outnumber macrophages in the shoulder region of lesions [33]. Intravital microscopy techniques demonstrate neutrophil rolling, arrest and transmigration of the vascular endothelium [33], [34]. Therefore, neutrophils not only adhere to the endothelium and transmigrate with the assistance of other inflammatory cells (e.g., mast cells, Figure 3), but may also activate the endothelium to recruit other leukocytes. Like macrophages, neutrophils produce inflammatory cytokines to induce MHEC adhesion molecule expression. Neutrophil lysates prepared from WT mice contained comparable levels of IL6, TNF-α, and IFN-γ to those in macrophage lysates (Table 1), and also showed significant enhancement of MHEC mRNA levels for all four tested adhesion molecules, as compared with those treated with medium alone (Figure 5A–5D). In contrast, lysates of neutrophils from *Tnf^−/−^* mice lost such activities, suggesting that neutrophils produce TNF-α to induce MHEC expression of P-selectin, E-selectin, VCAM-1, and ICAM-1. Neutrophils from *Ifng^−/−^* and *Il6^−/−^* exhibited different activities toward these four adhesion molecules. Similar to those from *Tnf^−/−^* mice, neutrophils from *Ifng^−/−^* mice also demonstrated reduced induction of ICAM-1, P-selectin, and E-selectin expression, but retained fully their level of activity in promoting VCAM-1 expression. In contrast, neutrophils from *Il6^−/−^* mice lost completely their ability to induce VCAM-1 expression, but retained the highest level of activity to induce ICAM-1, P-selectin, and E-selectin expression, among all three tested cytokine-deficient neutrophils evaluated (Figure 5A–5D). These observations show that neutrophil-derived TNF-α is a strong activator of adhesion molecule expression, whereas IL6 or IFN-γ activated selectively to induce certain adhesion molecules at mRNA levels. In contrast, neutrophil lysate-induced MHEC adhesion molecule protein levels showed different patterns from mRNA levels. While WT neutrophil lysates enhanced MHEC VCAM-1 protein levels, those from all three cytokine-deficient mice demonstrated significant reduction of VCAM-1 protein production (Figure 5E). E-selectin production in MHEC can be induced by neutrophil lysates prepared from both WT and *Tnf^−/−^* mice, but not those from *Il6^−/−^* or *Ifng^−/−^* mice (Figure 5F).

## Discussion

Mast cells, macrophages, neutrophils, and T-cells are among the most commonly observed inflammatory cells recruited to human and animal atherosclerotic lesions and in many other inflammatory diseases. Although dysregulated recruitment of these cells contributes to the pathogenesis of cardiovascular diseases, several important questions remain unsolved. Despite many reports of these cells producing “inflammatory mediators” that affect the endothelium lining the vessel wall and that these products promote leukocyte rolling, adhesion, and migration in the setting of atherosclerosis, obesity, or pulmonary infections, it remains unknown which cell type(s) cause the initial endothelial activation (i.e., adhesion molecule expression) and which cell types first transmigrate into the neointima during the development of atherosclerosis, or into the target tissues in other large vascular and microvascular diseases. These inflammatory cells produce many different cytokines, proteases, and other mediators. It is possible that only one particular set of such products participate in cell rolling, adhesion, and transendothelial migration. Furthermore, different inflammatory cells may produce the same cytokines for the same or different roles. This study, therefore, tested the role of three important secreted proinflammatory cytokines — TNF-α, IL6, and IFN-γ — from mast cells, macrophages, and neutrophils in primary microvascular MHEC activation. Our data indicate that all three cytokines, regardless of which cell type they originate, contributed to MHEC induction of VCAM-1, ICAM-1, P-selectin, or E-selectin expression. Among the three tested cytokines, TNF-α appeared the most important cytokine, regardless of cell sources or targets (adhesion molecules). IL6 and IFN-γ while less potent none-the-less were also critical and depended on the inflammatory cell type and the specific adhesion molecules. Although we did not test in this study the detailed molecular mechanisms by which these cytokines affect adhesion molecule expression, most likely these cytokines may act through their corresponding EC receptors. This hypothesis has been tested for mast cell–derived TNF-α. In mast cell–null mice, reconstitution with *Tnf^−/−^* mast cells showed a signaling defect that affects mast cell–EC interaction, whereas TNF-α receptor knockout (*Tnfr1^−/−^*) mice had substantially reduced mRNA and protein levels of P-selectin, E-selectin, ICAM-1, and VCAM-1 during delayed-type hypersensitivity response elicitation [35].

We showed previously that mast cell-derived TNF-α played negligible roles in atherosclerosis and abdominal aortic aneurysms (AAA) [23], [36]. Reduced atherosclerosis and AAA in mast cell-deficient *Kit^W-sh/W-sh^* mice can be fully restored by reconstituting these mice with mast cells from WT and *Tnf^−/−^* mice, but not those from *Il6^−/−^* or *Ifng^−/−^* mice, suggesting an essential role of mast cell IL6 and IFN-γ, but not TNF-α in atherosclerosis and AAA. However, the current study showed that mast cell TNF-α was not less potent than IL6 and IFN-γ in EC adhesion molecule expression and leukocyte recruitment (Figures 1–3). These observations suggest that TNF-α from other inflammatory cells (e.g. macrophages and neutrophils) compensate the loss of TNF-α in mast cells from *Tnf^−/−^* mast cell-reconstituted *Kit^W-sh/W-sh^* mice, leading to unaffected mast cell adhesion and recruitment. This hypothesis is consistent with the observations that mast cell numbers in *Kit^W-sh/W-sh^* mice receiving mast cells from WT mice or all three cytokine-deficient mice were comparable [23], [36].

One remaining question from our prior studies is why the same cytokines (IL6 or IFN-γ) from another inflammatory cells (e.g. macrophages, T cells, or neutrophils) did not compensate for the loss in mast cells during the pathogenesis of atherosclerosis and AAA [23], [36]. Here we confirmed that the same cytokines from different inflammatory cells acted differently in inducing MHEC adhesion molecule expression. In this study, macrophages from *Ifng^−/−^* mice showed a complete defect in VCAM-1 expression (Figure 4), but neutrophils from the same mice acted in the same way as WT neutrophils (Figure 5). Again, mast cells from *Il6^−/−^* mice showed a similar defect to that in mast cells from *Tnf^−/−^* mice in VCAM-1, ICAM-1, P-selectin, and E-selectin expression (Figure 1), but macrophages from the same mice showed no statistically significant differences from WT macrophages in ICAM-1 and E-selectin expression (Figure 4). One possibility is that the lack of one cytokine affects the expression of the other cytokines; the defects observed from one cytokine-deficient cell, therefore, might be combinational defects of multiple mediators associated with this cytokine. For example, we have demonstrated that mast cells from *Il6^−/−^* mice had low levels of TNF-α and IFN-γ, and vice versa [23]. Although not tested, a lack of the same cytokine may affect different sets of other cytokines or other mediators in different inflammatory cells. For example, mast cells from *Il6^−/−^* mice had low IFN-γ and TNF-α, but the same may not necessarily be true in macrophages or neutrophils from *Il6^−/−^* mice. Further investigations are required to test this hypothesis.

Here we tested the function of common cytokines from mast cells, macrophages, and neutrophils in several well-known adhesion molecules. However, this study has its limitations. For examples, other inflammatory cells, such as T-cells [37] and eosinophils [38], also appear in human atherosclerotic lesions. These cells may also produce TNF-α, IFN-γ, IL6, and many other cytokines for endothelium activation, or other molecules to assist leukocyte adhesion and transmigration. Furthermore, TNF-α, IFN-γ, IL6 are not the only important pro-inflammatory cytokines of mast cells, macrophages, or neutrophils. Many other untested cytokines (e.g. IL1, IL4), chemokines (e.g. MCP-1, IL8), proteases (e.g. neutrophil elastase, tryptase, cysteinyl cathepsins), and other inflammatory mediators (e.g. histamine, substance P, and angiotensin-II) may also contribute to endothelium activation via various mechanisms. Nevertheless, our study provides the first comparisons of different inflammatory cell types from the same mice to act on the same MHEC, but exhibit different activities in MHEC adhesion molecule expression.
